# Endogenous Extracellular Vesicles Participate in Brain Remodeling after Ischemic Stroke

**DOI:** 10.3390/ijms242316857

**Published:** 2023-11-28

**Authors:** Mauricio Muleiro Alvarez, Felipe Esparza Salazar, Thomas Rodriguez, Francesco D’Egidio, Cesar V. Borlongan, Jea-Young Lee

**Affiliations:** Center of Excellence for Aging and Brain Repair, Department of Neurosurgery and Brain Repair, Morsani College of Medicine, University of South Florida, 12901 Bruce B. Downs Blvd., Tampa, FL 33612, USA; mauricio.muleiroal@anahuac.mx (M.M.A.); felipe.esparzas69@anahuac.mx (F.E.S.); trodriguez862@gmail.com (T.R.); francescodegidio@usf.edu (F.D.); jeayoung@usf.edu (J.-Y.L.)

**Keywords:** cerebral ischemia, exosomes, neuroprotection, glial activation, neurogenesis, angiogenesis

## Abstract

Brain remodeling after an ischemic stroke represents a promising avenue for exploring the cellular mechanisms of endogenous brain repair. A deeper understanding of these mechanisms is crucial for optimizing the safety and efficacy of neuroprotective treatments for stroke patients. Here, we interrogated the role of extracellular vesicles, particularly exosomes, as potential mediators of endogenous repair within the neurovascular unit (NVU). We hypothesized that these extracellular vesicles may play a role in achieving transient stroke neuroprotection. Using the established ischemic stroke model of middle cerebral artery occlusion in adult rats, we detected a surged in the extracellular vesicle marker CD63 in the peri-infarct area that either juxtaposed or co-localized with GFAP-positive glial cells, MAP2-labeled young neurons, and VEGF-marked angiogenic cells. This novel observation that CD63 exosomes spatially and temporally approximated glial activation, neurogenesis, and angiogenesis suggests that extracellular vesicles, especially exosomes, contribute to the endogenous repair of the NVU, warranting exploration of extracellular vesicle-based stroke therapeutics.

## 1. Introduction

Ischemic stroke accounts for almost 80% of all strokes worldwide and is also the leading cause of disability and the second leading cause of global deaths [1]. However, therapeutic options are very limited and include endovascular treatment or intravenous thrombolysis with tissue plasminogen activator, known as tPA [2]. These options are highly limited due to the extremely narrow therapeutic window for tPA use (4.5 h) and very strict selection criteria. As a result, nearly 90% of patients suffering from an ischemic stroke cannot access the treatment [3].

After the ischemic event, inflammatory cascades occur, which exacerbate the neurological damage caused by the stroke. This is observed in both the central core of the ischemia and the ischemic penumbra area [4,5]. There are different mechanisms of cell death that exacerbate the injury itself, along with secondary damage [6,7], such as the accumulation of free radicals, abnormal vasculogenesis, oxidative stress, decreased neurogenesis and angiogenesis, mitochondrial dysfunction, and excitotoxicity [8,9]. Furthermore, a structure that plays a fundamental role in achieving proper recovery after an ischemic stroke is the neurovascular unit (NVU). The NVU is a structure composed of different components such as neurons, astrocytes, and endothelial cells, among others, which have a direct interaction between cerebral vasculature and neurons [10]. Another primary function of the NVU is to regulate cerebral blood flow along with the blood–brain barrier (BBB) to maintain adequate homeostasis [11]. An increase in BBB permeability and dysfunction of the NVU result in greater hemorrhage, edema, and deleterious peripheral inflammatory cell infiltration of the ischemic brain, altogether worsening the stroke outcomes [12].

Clearly, there is an urgent need to develop new treatments that do not solely target the acute primary injury phase of ischemic stroke but also effectively sequester the secondary cell death due to stroke. One of the therapeutic strategies that has been under investigation in recent decades is the use of stem cells, such as mesenchymal stem cells, hematopoietic stem cells, and neural progenitor cells [2]. The mobilization of endogenous stem cells towards the injured host brain may render functional benefits via cell replacement and by-stander effects (e.g., neurotrophic factor secretion) [13,14]. Recently, the secretion of extracellular vesicles by stem cells has been postulated as an equally robust brain repair process [15,16]. Extracellular vesicles are capable of mediating cellular communication through the secretome released into the extracellular space [17,18,19], where they contain components of cellular signaling, cytokines that can modify the extracellular matrix, as well as influence cell differentiation and proliferation, growth factors, and, in recent studies, gene modifications [20,21,22,23,24]. Identifying the specific extracellular vesicles or exosomes that mediate the therapeutic effects of stem cells remains underexplored.

**Figure 1:**
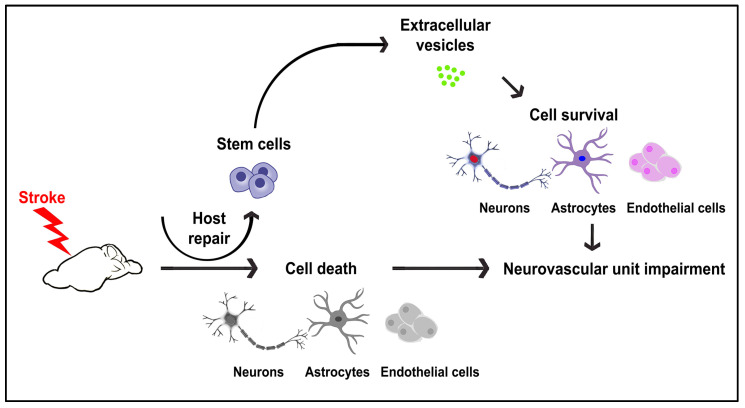
Extracellular vesicle-mediated neuroprotection. A potential host repair mechanism involves the upregulation of endogenous extracellular vesicles contributing to the repair of the NVU after stroke.

The utility of stem cells as a treatment for ischemic stroke has been demonstrated, both in experimental studies and clinical cases. Undoubtedly, clarifying the cellular mechanisms by which stem cells afford therapeutic effects will allow the optimization of safety and efficacy of their clinical application for stroke patients. Here, we advance the concept of extracellular vesicles, in particular exosomes, as possibly mediating the endogenous repair of the NVU, albeit transient stroke neuroprotection (Figure 1).

## 2. Results

### 2.1. Infarct Volumes and Peri-Infarct Areas

**Figure 2:**
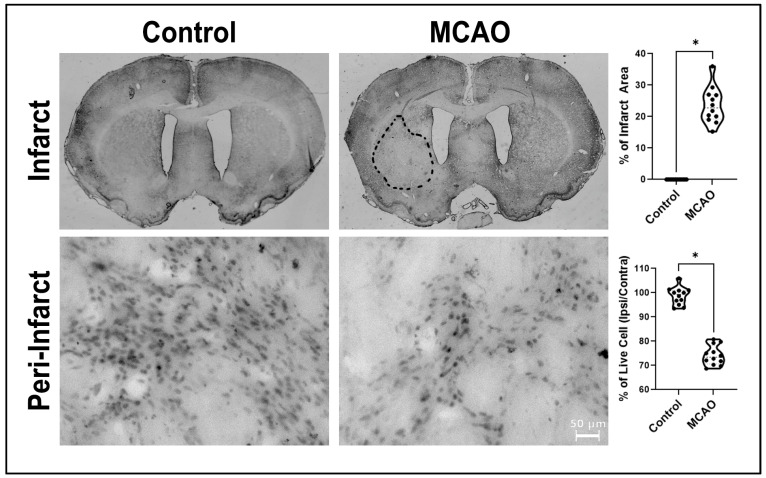
Infarct volume and peri-infarct area. The ischemic stroke model of MCAO produced the typical striatal and cortical infarction with a peri-infarct area adjacent to this region. Neither infarcts nor peri-infarct area was visible in control animals. *p* value * corresponds to 0.0001. Scale bar = 50 μm.

Animals in the control group showed no detectable infarcts with intact brain tissue whereas animals in the MCAO group displayed the typical tissue infarction in the cortex and striatum (* *p* < 0.0001, F_11,11_ = 22.74) (Figure 2). Additionally, quantitative analysis of the peri-infarct area cell count revealed that control animals displayed a significantly greater percentage of viable cells (* *p* < 0.0001, F_11,11_ = 1.248) compared to the MCAO animals (Figure 2).

### 2.2. Glial Activation and Extracellular Vesicle Expression in the Stroke Brain

**Figure 3:**
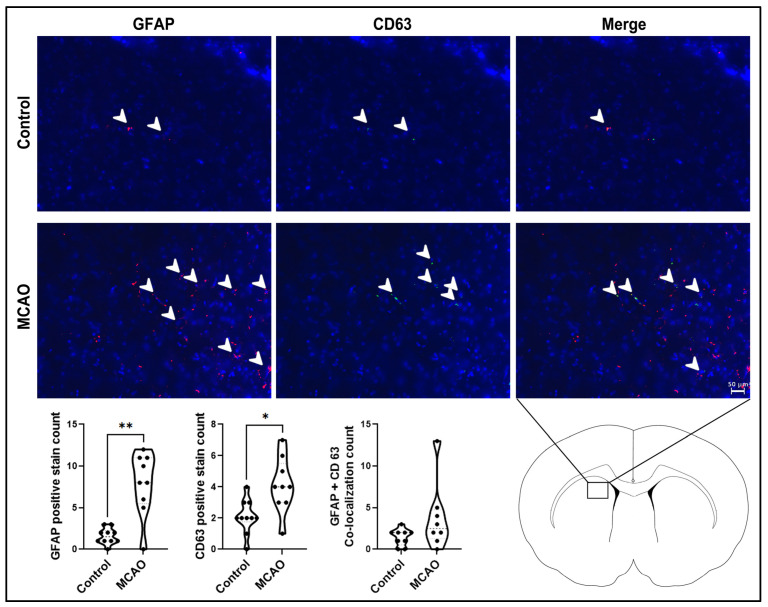
Glial activation and extracellular vesicle expression in the stroke brain. The glial marker GFAP and the extracellular vesicle marker CD63 revealed significantly upregulated glial activation and exosome content within the peri-infarct area of MCAO animals compared to control animals. White arrow heads point to GFAP-positive stains. *p* values * and ** correspond to 0.01 and 0.0001, respectively. Scale bar = 50 μm.

The glial marker GFAP and the extracellular vesicle marker CD63 (Figure 3) were used to analyze the glial activation and exosome content within the peri-infarct area. Both markers showed significant elevations in the MCAO group (** *p* < 0.0001, F_8,9_ = 15.39), CD63 (* *p* < 0.01, F_8,9_ = 2.569), and double-labeled GFAP and CD63 (*p* = 0.1193, F_7,8_ = 16.05) compared to the control group, demonstrating increased GFAP-positive glial cells juxtaposed to CD63-labeled exosomes.

### 2.3. Neurogenesis and Extracellular Vesicle Expression in the Stroke Brain

**Figure 4:**
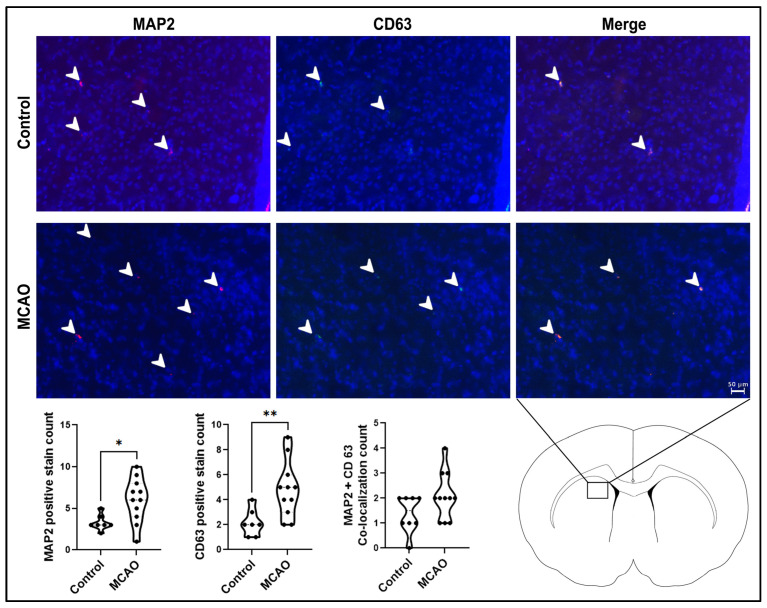
Neurogenesis and extracellular vesicle expression in the stroke brain. The neurogenic marker MAP2 and the extracellular vesicle marker CD63 showed significant increased expression in MCAO animals compared to control animals. White arrow heads point to MAP2-positive stains. *p* values * and ** correspond to 0.001 and 0.01, respectively. Scale bar = 50 μm.

The neurogenic marker MAP2 and the extracellular vesicle marker CD63 (Figure 4) were used to analyze neurogenesis and extracellular vesicle expression in the peri-infarct area. Both markers showed a significant increase in the MCAO group (* *p* < 0.001, F_11,11_ = 21.69), CD63 (** *p* < 0.01, F_10,6_ = 4.343), and double-labeled MAP2 and CD63 (*p* = 0.0935, F_10,7_ = 1.609) compared to the control group, showing that MAP2-labeled young neurons populated the ischemic region adjacent to CD63-labeled exosomes.

### 2.4. Angiogenesis and Extracellular Vesicle Expression in the Stroke Brain

**Figure 5:**
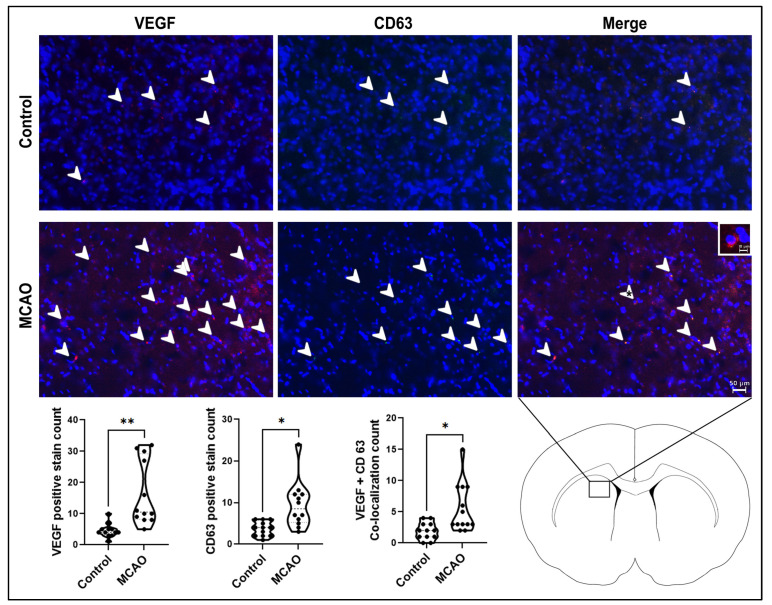
Angiogenesis and extracellular vesicle expression in the stroke brain. The angiogenic marker VEGF and the extracellular vesicle marker CD63 displayed significant elevations in MCAO animals compared to control animals. Small white box showed zoom in picture for VEGF and CD63 co-localized cell. White arrow heads point to VEGF-positive stains. *p* values * and ** correspond to 0.01 and 0.001, respectively. Scale bar = 50 μm and 10 μm.

The angiogenic marker VEGF and the extracellular vesicle marker CD63 (Figure 5) were used to analyze the angiogenesis and extracellular vesicle expression in the peri-infarct area. Both markers showed significant increases in the MCAO group (* *p* < 0.01, F_10,6_ = 7.737), CD63 (** *p* < 0.001, F_11,11_ = 10.36), and double-labeled VEGF and CD63 (** *p* < 0.001, F_12,11_ = 8.030) compared to the control group, indicating that VEGF-labeled angiogenic cells co-localized with CD63-labeled exosomes.

## 3. Discussion

Host brain repair ensues during the acute stage of stroke, but without any exogenous treatment intervention the cascade of secondary cell death mechanisms commences, exacerbating the pathological symptoms of stroke. Thus, enhancing endogenous neuroprotection may allow a wider therapeutic window for exogenous treatment while retarding and even halting the secondary cell death. To this end, however, tPA and mechanical thrombectomy only target the acute primary injury. Safe and effective novel therapies are needed to address the secondary cell death of stroke. Here, we showed that acutely, extracellular vesicles were upregulated within the peri-infarct area of the stroke brain, which appeared to accompany endogenous neuroprotective processes, including glial activation, neurogenesis, and angiogenesis. That the expression of extracellular vesicles spatially and temporally approximated host reparative mechanisms suggests that treatment strategies directed at amplifying extracellular vesicles post-stroke may render neuroprotective, as well as regenerative effects on the stroke brain.

Exosomes are extracellular vesicles with a diameter of 30–100 nm. They contain DNA, proteins, mRNA, and some non-protein-coding RNAs, and are secreted by different cells, including endothelial cells. In particular, exosomes derived from brain microvascular endothelial cells have demonstrated protective effects in counteracting apoptosis and promoting synaptic remodeling in stroke mice, supporting the idea of the contribution of endothelial cells in the NVU microenvironment in stroke [25]. Exosomes are responsible for transporting transductive information between cells for the transduction of information. Exosomes from mesenchymal stem cells derived from adipose tissue exert a protective role against nerve injuries due to glutamate and cell cycle arrest caused by ischemic injury [26,27]. Among the many neurovascular factors secreted by mesenchymal stem cells [28,29,30,31,32,33], CD63-expressing exosomes present in lysosomal membranes exert platelet activity; in ischemic stroke they promote platelet hyperactivation [34].

The presence of exosomes in the brain has been recognized largely due to the abundance of CD63, among other markers, within the exosomes, implicating their involvement in brain cell-to-cell interactions [35,36]. These findings support the idea of exosomes as a highly relevant player in the interplay between endogenous extracellular vesicles and NVU repair. Regarding the choice of CD63 as one of the exosomal markers, in vitro and in vivo studies have consistently detected CD63 among analyzed exosomes. For instance, a recent study [37] shows that extracellular vesicles derived from human plasma reflect the cell of origin of disease prognosis and severity. Interestingly, flow cytometry revealed that individual neuronal extracellular vesicles isolated from plasma samples can be identified using size exclusion chromatography and that, among other results, the extracellular vesicles are mainly (95%) CD63+ exosomes of endosomal origin [37]. In another study, exosomes derived from neural stem cells used in combination with neural stem cells to treat stroke mice exert neuroprotective effects [38]. The exosomes administered were positive for TSG101 and CD9, but in particular for CD63 [38]. The present study focused on CD63, but other surrogate exosomal markers, such as TSG101 and CD9, can be employed in future studies.

CD63 is reported to be elevated in atherosclerotic ischemic stroke in the acute phase [39,40,41], suggesting that increased CD63 on platelets reveals acute damage. This is because the thrombi that cause stroke occur mainly in the large vessels and due to the inflammatory process. Platelet expression of CD63 remains elevated even 90 days after the atherosclerotic ischemic stroke [38,42]. CD63 is exposed on the surface of activated platelets, and CD63 on platelets is reported to be increased in atherosclerotic ischemic stroke and in some cases persists chronically [40]. CD63 on platelets has inflammatory activity and participates during atherogenesis by destabilizing the atheroma of the vertebrobasilar and carotid arteries. This destabilization is caused by inflammatory cytokines and activated T cells that activate macrophages to degrade the matrix causing thromboembolism and acute ischemic events. In the acute phase there is greater platelet activation, increasing the CD63 marker [40,43,44,45]. In the clinic, CD63 is significantly higher in patients with acute stroke than in control subjects [46]. Both laboratory and clinical studies suggest that extracellular vesicles, including exosomes, may participate in brain homeostasis and disease states.

The present observations that CD63-labeled exosomes were upregulated after stroke may signal a neuroprotective mechanism. Indeed, in discreet regions of the peri-infarct area, CD63 expression was juxtaposed with glial activation, neurogenesis, and angiogenesis. Glial activation is a double-edged sword, whereby its acute expression may be beneficial [47], but when it occurs chronically, then such gliosis may exacerbate stroke outcomes [48]. Moreover, extracellular vesicles have been implicated in the neuroprotective function of glial cells [49]. In a similar fashion, both neurogenesis and angiogenesis are established neuroprotective and regenerative mechanisms for stroke, especially in the repair of the NVU [50,51,52], with extracellular vesicles becoming widely recognized as contributing to these therapeutic responses [53,54,55]. Interestingly, CD63 expression was shown here to be significantly pronounced and co-localized with angiogenic marker VEGF compared to glial marker GFAP or neurogenic marker MAP2, suggesting that the CD63-labeled extracellular vesicles preferably mediated host angiogenesis after stroke. 

## 4. Materials and Methods 

### 4.1. Subjects

**Figure 6:**
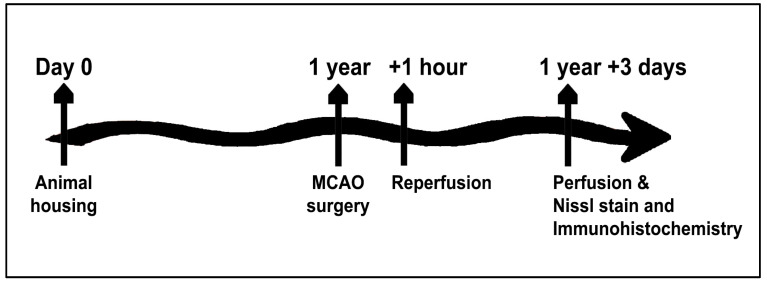
Time Line. One year old rodents were subjected to MCAO then 3 days later examined for stroke outcomes and mechanistic participation of extracellular vesicles in the endogenous repair processes of glial activation, neurogenesis, and angiogenesis.

The experiment was approved by the Institutional Animal Care and Use committee of the University of South Florida, Morsani College of Medicine (Approval Code: IS00009075, Cell-Based Regenerative Medicine for Stroke; Approval Date: 5/3/2021 and Expiration Date: 4/30/2024) and was conducted in accordance with the National Institute of Health Guide and Use of Laboratory Animals. All animals were housed in a 12 h light/dark cycle at 20 °C, 50% relative humidity with free access to food and water. A total of 8 adult male Sprague–Dawley rats (1 year old, 400–450 g) were used for this study (Figure 6).

### 4.2. Stroke Surgical Procedure

Adult Sprague–Dawley rats were subjected to stroke (n = 4) or sham surgery (n = 4) and were anesthetized using a mixture of 1–2% isoflurane in nitrous oxide/oxygen (69%/30%) using a facial mask. Body temperature was maintained at 37 ± 0.3 °C during the surgical procedure. A middle skin incision was made on the neck to access and examine the right common carotid artery (CCA), the internal carotid artery, and the external carotid artery. A single 4-0 monofilament nylon suture (27.0–28.0 mm) was carefully threaded from the point where the CCA divided all the way to obstruct the middle cerebral artery (MCA). The animals were given time to awaken from anesthesia after the middle cerebral artery occlusion (MCAO) procedure. Following 60 min of temporary MCAO, the animals were sedated again, and the nylon thread was removed to allow reperfusion. For the sham surgery group, animals were anesthetized, and a central incision was made on the neck to expose and isolate the right CCA. The incision was then closed, and the animals were allowed to recover from anesthesia.

### 4.3. Perfusion

Under deep anesthesia, the rats were euthanized on the third day of the experiment for immunofluorescence. For the immunofluorescent analysis, a cold phosphate-buffered solution (PBS) was initially perfused through the ascending aorta at a volume of 250–300 mL, followed by a perfusion of 250–300 mL of 4% paraformaldehyde in phosphate buffer (PB). Subsequently, the brains were carefully extracted and post-fixed using the same paraformaldehyde solution for a duration of 48 h. After the post-fixation, the brains were transferred to a solution of 30% sucrose in PB until they fully sank. Coronal sections, each with a thickness of 40 µm, were meticulously obtained using a cryostat and were stored at a temperature of −20 °C for further analysis.

### 4.4. Nissl Staining

Nissl staining was performed with 0.1% cresyl violet solution using a standard protocol to evaluate the peri-infarct injury of our MCAO model. Eight coronal sections between the anterior edge and posterior edge of the MCAO infarct area were collected and processed for Nissl staining from each perfused brain. One in every eight coronal tissue sections was chosen at random to quantify cell survival in the peri-infarct area. Brain sections were examined using a light microscope. Neuronal survival in the peri-infarct area was quantified using a computer-assisted image analysis system and was expressed as a percentage of the ipsilateral hemisphere compared to the contralateral hemisphere. The infarction area ratio was calculated by defining left hemisphere (LT), right hemisphere (RT), and infarction area (RI) in mm^2^. Infarction area ratio = [LT − (RT − RI)] × 100/LT (%) [56].

### 4.5. Immunohistochemistry Protocol

Brain sections were processed for mechanism-based immunohistochemical analyses focusing on inflammation. Immunofluorescence labeling was conducted on every fourth coronal section of the brain. Sections were washed three times in 0.1 PBS for 10 min. Six sections were incubated with saline sodium citrate solution at pH 6.0 for 40 min at 80 °C for antigen retrieval purposes. After, samples were blocked for 60 min at room temperature with 8% normal goat serum in 0.1 PBS containing 0.1% Tween 20. Then, sections were incubated overnight at 4 °C with corresponding primary antibody with 10% normal goat serum. The primary antibodies used for brain tissue were rabbit polyclonal GFAP (1:500; abcam, Waltham, MA, USA), MAP2 (1:500; abcam, Waltham, MA, USA), VEGF (1:500; abcam, Waltham, MA, USA), and mouse monoclonal CD63 (1:500; Novusbio, Centennial, CO, USA). Sections were washed for 10 min in 0.1 PBS containing 0.1% Tween 20 five times and soaked in 5% normal goat serum in 0.1 PBS containing corresponding secondary antibodies, goat anti-mouse IgG-Alexa 488 (green) (1:500; Invitrogen) or goat anti-rabbit IgG-Alexa 594 (red) (1:500; Invitrogen, Waltham, MA, USA) for 2 h. Sections were washed at least three times for 10 min in 0.1 PBS, cover-slipped with Vectashield with DAPI (H-1500). The sections were examined using a confocal microscope (Zeiss, Dublin, CA, USA).

### 4.6. Statistical Analysis

Data were statistically analyzed using one-way analysis of variance (ANOVA) followed by post hoc Bonferroni’s test. Statistical significance was presented at *p* < 0.05 (GraphPad version 9). The Kolmogorov–Smirnov test was used to assess normality and the resulting values were <5% of the critical values [57].

## 5. Conclusions

To date, treatments for ischemic stroke have focused on the use of tPA and mechanical thrombectomy, generally designed to manage the consequences of acute primary injuries. However, there are currently no therapeutic solutions to the problem of secondary cell death after stroke. To this end, the development of safe and effective strategies targeting the stroke secondary cell death is necessary. The present study reveals a close interaction between extracellular vesicles and endogenous brain repair mechanisms offering a fertile ground for the development of extracellular vesicle-based stroke therapeutics. Furthermore, future experiments will help to further analyze exosomes in terms of exosome markers and content, providing the possibility to optimize protocols and experimental conditions [58].

## Data Availability

Data are contained within the article.
